# Frequency and characterization of β-lactam-resistant *Escherichia coli* from wild birds from Marmara Region in Turkey

**DOI:** 10.1007/s42770-026-01941-9

**Published:** 2026-05-06

**Authors:** Barış Halaç, Ayşe Ilgın Kekeç, Baran Çelik, Deniz Kesen, Beren Başaran Kahraman, Belgi Diren Sığırcı, Arzu Funda Bağcıgil, Kemal Metiner, Yavuz Çokal, Seyyal Ak

**Affiliations:** 1 Department of Microbiology, Faculty of Veterinary Medicine, Istanbul University - Cerrahpaşa, Istanbul, Turkey; 2 Institue of Graduate Students, Istanbul University - Cerrahpaşa, Istanbul, Turkey; 3 Bandirma Vocational School, Bandirma Onyedi Eylül University, Balikesir, Turkey

**Keywords:** Antbiotic resistance, Beta lactam, *Eschericia coli*, Wild birds, One health

## Abstract

This study aimed to phenotypically and genotypically detect beta-lactam-resistant *Escherichia coli* in the feces of wild and migratory birds residing in or passing through the Marmara Region during seasonal migration. A total of 272 fresh fecal samples were non-invasively collected and grouped as follows: (1) resident wild waterfowl, (2) resident urban birds (e.g., pigeons, crows, sparrows), (3) migratory birds overwintering in Turkey, and (4) migratory birds staying during spring and summer. Samples were cultured on MacConkey agar with and without cefotaxime (1 mg/L). Carbapenem resistance was assessed using the Modified Hodge Test; none of the 212 isolates tested positive. Among the 272 birds sampled, 62 (22.8%) carried ESBL and/or AmpC producing isolates. Antimicrobial-resistant *E. coli* were identified in 59 of 272 birds (21.7%), including 47 ESBL-positive, 6 AmpC-positive, and 5 harboring both resistance types. Group 1 (mainly gulls) showed the highest resistance rates. PCR analysis revealed that among 84 isolates, 48 carried only beta-lactamase genes, 3 only AmpC genes, and 5 both. Detected genes included: *bla*_CTX-M_ (*n* = 50), *bla*_SHV_ (*n* = 2), and *bla*_OXA10_ (*n* = 10); for AmpC: *bla*_MOX_ (*n* = 6) and *bla*_CIT_ (*n* = 2). Species-specific results confirmed the highest frequency of resistance genes in gulls. The Frequency of resistant isolates was significantly higher in Groups 1 and 2 (*P* < 0.001). The resistance profiles identified in gulls especially those feeding at landfills or in direct contact with human, animal, and agricultural waste suggest a likely anthropogenic and/or zoonotic origin of the beta lactam resistance observed in this study conducted in Marmara Region.

## Introduction

The widespread use of antimicrobial agents has led to significant advances in the treatment of infectious diseases in both human and veterinary medicine. However, the growing global population, intensive animal production systems, and the uncontrolled or inappropriate use of antibiotics have accelerated the emergence and global spread of antimicrobial resistance. Today, resistant bacteria are detected not only in clinical settings but also in ecosystems associated with the environment and wildlife; their presence has even been demonstrated in remote regions such as the Peruvian Amazon [1]. This situation demonstrates that antimicrobial resistance cannot be explained solely by direct antibiotic exposure but is a complex ecological process shaped by interactions among humans, animals, and the environment.

In recent years, wild birds have increasingly drawn attention as reservoirs of antimicrobial-resistant bacteria and potential vectors in their spread. Due to their high mobility, diverse feeding habits, and frequent contact with anthropogenic sources, these birds can acquire resistant microorganisms from environments such as agricultural areas, landfills, wastewater systems, and contaminated water sources [2–4]. These microorganisms and their associated resistance genes can be transported across wide geographic areas through the birds’ migrations and daily movements and can circulate among human, domestic animal, and other wildlife populations [5, 6]. However, the true epidemiological role of wild birds in this process has not yet been fully elucidated.

Studies conducted in different geographic regions have demonstrated the presence of Enterobacterales producing GSBL and/or AmpC in wild birds, particularly those associated with urban and aquatic ecosystems. However, reported prevalence rates and the distribution of resistance genes show significant variations depending on regional environmental conditions, bird species, and the level of anthropogenic impacts. Furthermore, a significant portion of existing studies has focused solely on phenotypic resistance profiles or a limited number of genotypic markers, and the number of comprehensive studies evaluating both phenotypic and genotypic data together remains relatively limited. Furthermore, the majority of data in the literature has been obtained from specific regions of Western Europe and Asia, and there remains a lack of data for regions with high ecological diversity and strategic geographic locations [7–11].

Although the presence of bacteria producing GSBLs in Turkey has been demonstrated in various animal groups, particularly farm animals, data regarding wild bird populations are limited [12, 13]. This situation is all the more significant given that Turkey lies along major migration routes between Europe, Asia, and Africa. In particular, the Marmara Region serves as a crucial stopover, feeding, and breeding ground for many resident and migratory bird species, thanks to its wetland ecosystems. The interaction of these birds with dense human populations, agricultural activities, and coastal ecosystems creates a notable environment for the environmental circulation of antimicrobial resistance determinants.

In this context, the limited number of studies evaluating resistance traits at both the phenotypic and genotypic levels in resident and migratory wild bird populations in the Marmara Region indicates a significant knowledge gap. Such studies will not only address the regional data gap but also contribute to a more comprehensive assessment within the One Health framework, which addresses the interactions of antimicrobial resistance among humans, animals, and the environment.

## Materials and methods

### Study design

A total of 272 fresh fecal samples were collected noninvasively from resident and migratory wild birds in the Marmara Region of Turkey. The sample size for the study was determined based on a 50% estimated prevalence, a 90% confidence level, and 5% absolute certainty. The samples collected in the study were grouped according to whether the wild birds were resident or migratory, as well as according to their migration periods. In addition, the ecological characteristics of the bird species and their degree of anthropogenic environmental exposure were taken into consideration in this classification. The grouping was structured based on the likelihood of wild birds inhabiting different environments coming into contact with humans or domestic/wild animals. The birds were classified into four groups, each consisting of 68 samples, as outlined in Table 1; Group 1: Resident wild waterbirds (e.g., great cormorants -*Phalacrocorax carbo*, gulls -*Larus spp.*, grey herons-*Ardea cinerea*) captured near wetland areas in proximity to human activity. Group 2: Urban-resident terrestrial birds (e.g., doves, pigeons -*Columbidae*, crows -*Corvus corone*, sparrows -*Passer domesticus*). Group 3: Winter migratory birds that remain in the region through February. Group 4: Summer migratory birds that arrive during the spring season. This grouping was designed to enable comparative analysis of beta lactam resistance patterns among birds with varying degrees of environmental exposure and migratory behavior.

**Table 1:** The species and subspecies wise distribution of the collected fecal samples

Groups	Species/Subspecies		Number of birds		Total number
Group 1	Seagull	Little Gull	8	59	68
		Herring Gull	51		
	Grey Heron		3		
	Gaunt		1		
	Bittern		4		
	Water Rail		1		
Group 2	Pigeon		38		68
	Dove		12		
	Crow		12		
	Magpie		6		
Group 3	Falcon		16		68
	Hawk		10		
	European honey buzzard		8		
	Night Peck		6		
	Owl	Tawny Owl	1	14	
		Eared Owl	2		
		Woodcock Owl	5		
		Silver Owl	6		
	Wind-sucker		4		
	Short-toed Eagle		2		
	Owling		5		
	Alpine Swift		2		
	Hobby		1		
Group 4	Starling		24		68
	Pygmy Cormorant		14		
	Stork		5		
	Dalmatian Pelican		6		
	Finch		2		
	Squacco Heron		12		
	Glossy Ibis		4		
	Chimney Swallow		1		

Fecal sampling from wild avian populations was conducted using non-invasive techniques to ensure minimal physiological distress. Fresh fecal samples were collected from birds that had been admitted to private veterinary clinics and to the Wildlife Rehabilitation Center of Istanbul University-Cerrahpaşa Faculty of Veterinary Medicine for examination, care, rehabilitation, or treatment. Samples were obtained immediately after defecation during these procedures. Additionally, fecal samples were collected from birds defecating within large flocks congregating in wooded areas located in various districts of Istanbul Province. At the Manyas Bird Paradise, fresh droppings of wild waterbirds were collected under the supervision of responsible veterinarians by following the birds as they came ashore. In the coastal zones of the Marmara Region, fecal samples were also obtained from aquatic birds nesting on rooftops of commercial buildings, with collections performed immediately after defecation. All fecal samples were transported to the laboratory using Cary-Blair transport swabs.

### Bacterial isolation and identification

Fecal samples were incubated aerobically at 37 °C for 24 h in both cefotaxime-supplemented (1 mg/L) and plain MacConkey agar. The use of cefotaxime-supplemented MacConkey agar has been reported by EFSA to enhance the success of primary isolation [12–14]. Colonies with typical *E. coli* morphology were identified and confirmed using standard biochemical tests. More than one isolate per sample was allowed [15].

### Phenotypic detection of ESBL, AmpC beta-lactamase, and carbapenemase-producing *E. coli*

*E. coli* isolates from cefotaxime-supplemented MacConkey agar were screened for ESBL and AmpC beta-lactamases. Usually, a single isolate was collected for each bird. However, for birds yielding morphologically diverse colonies, samples were divided into “a” and “b” subsets to distinguish different isolates. ESBL production was initially screened using sensitivity to cefpodoxime, ceftazidime, aztreonam, cefotaxime, and ceftriaxone [16]. Confirmation was performed via disk diffusion using cefotaxime (30 µg), ceftazidime (30 µg), and both in combination with clavulanic acid (30/10 µg) [17]. AmpC beta-lactamase was inferred in isolates resistant to cefoxitin (≤ 14 mm) and susceptible to cefepime (≥ 18 mm) [18]. Additionally, carbapenemase production was assessed using the Modified Hodge Test on isolates grown on MacConkey agars without cefotaxime [16].

### Beta lactam resistance genes in *E. coli* isolates

The presence of ESBL and AmpC beta-lactamase resistance genes (*bla*_TEM_, *bla*_CTX-M_, *bla*_SHV_, *bla*_CMY_, *bla*_OXA-10_, *bla*_PER-2_, *bla*_CIT_, *bla*_DHA_, *bla*_MOX_, *bla*_FOX_, *bla*_EBR_, and *bla*_ACC_) was investigated via conventional PCR in all *E. coli* strains isolated from MacConkey agar supplemented with cefotaxime, regardless of phenotypic findings. Conversely, the presence of carbapenemase resistance genes (*bla*_KPC_, *bla*_VIM_, *bla*_OXA_, *bla*_NDM-1_) was screened in *E. coli* strains isolated from non-supplemented MacConkey agar [18, 19]. All genes and cycling conditions summarized in supplementary file. DNA extraction from *E. coli* isolates was performed via the boiling method according to Sigirci et al. [18]. One loopful of each 24-hour fresh culture was homogenized in 100 µl of DNase/RNase-free ultrapure water. Following a 10-minute incubation at 95 °C in capped microcentrifuge tubes, the samples were centrifuged at 6000 rpm for 4 min. The harvested supernatant was then used as the DNA template in subsequent PCR assays.

### Data analysis

Descriptive statistics for the variables examined in the study were expressed as absolute frequencies (n) and relative frequencies (%). Prevalence rates (PR) for antimicrobial resistance were calculated separately for phenotypic and genotypic resistance. Prevalence was determined by dividing the number of antimicrobial-resistant *E. coli* isolates by the total number of *E. coli* isolates examined. All statistical analyses in this study were performed at the isolate level. Confidence intervals for prevalence rates were calculated at a 90% confidence level (90% CI). The primary variables evaluated in the analyses were defined as the presence of rectal colonization, phenotypic antibiotic resistance, genotypic antibiotic resistance, and the group variable to which the samples belonged. The rectal colonization variable was characterized by the isolation of *E. coli* from rectal samples obtained from wild birds and the subsequent determination of the antibiotic resistance profiles of these isolates. Within this framework, the relationship between rectal colonization and the presence of antibiotic resistance was evaluated. Initially, the total antibiotic resistance prevalence was calculated for all samples. Subsequently, phenotypic and genotypic antibiotic resistance prevalences were determined for each study group individually. Pairwise comparisons were conducted between the prevalence values of the groups to evaluate inter-group differences. Chi-square ($\chi^2$) tests were employed to compare categorical variables. In cases where the expected cell frequency was less than 5, Fisher’s exact test was applied. To mitigate the risk of Type I errors arising from multiple group comparisons, a Bonferroni correction was implemented. For all analyses, a p-value of < 0.05 was considered statistically significant. All statistical analyses were performed using SPSS for Windows Version 30.0 (SPSS Inc., Chicago, IL, USA).

## Results

Of 272 specimens, 212 and 84 *E. coli* colonies were isolated and identified from plain and cefotaxime-supplemented MacConkey agar, respectively. The group-wise distribution of the identified *E. coli* isolates is summarised in Table 2.

### Data regarding phenotypically detected ESBL, AmpC, and carbapenemase activities

Of the 84 *E. coli* isolates resistant to cefotaxime, 55 (65.47%) had phenotypic ESBL activity and 12 (14.28%) had AmpC activity. Of these *E. coli* strains, 50 had ESBL alone, 7 had AmpC alone and 5 had both ESBL and AmpC activities. The total number of ESBL and/or AmpC positive isolates was 62/272 (22.8%) (Table 2).

**Table 2:** The group-wise distribution of the phenotypically characterized ESBL and/or AmpC-producing *E. coli* isolates

Groups (*n*)	Fenotypic Results						Genotypic Results of ESBL				Genotypic Results of AmpC	
	CMCA*	ESBL-positive*	AmpC- positive*	ESBL and AmpC- positive*	ESBL positive per se*	AmpC positive per se*	CTX-M	SHV	OXA 10		CIT	MOX
Group 1 *n* = 68	48/68	32/48 (66.7%)	9/48 (18.75%)	4/48 (8.3%)	28/48 (58.3%)	5/48 (10.42%)	25/48 (52,08%)	1/48 (2,08%)	6/48 (12,5%)		1/48 (2,08%)	5/48 (10,41%)
Group 2 *n* = 68	20/68	13/20 (65%)	1/20 (5%)	-	13/20 (65%)	1/20 (5%)	12/20 (60%)	1/20 (5%)	1/20 (5%)		-	1/20 (5%)
Group 3 *n* = 68	10/68	8/10 (80%)	-	-	8/10 (80%)	-	5/10 (50%)	-	2/10 (20%)		-	-
Group 4 *n* = 68	6/68	2/6 (33.3%)	2/6 (33.3%)	1/6 (16.6%)	1/6 (16.6%)	1/6 (16.6%)	5/6 (83,3%)	-	1/6 (16,6%)		1/6 (16,6)	-
Total *n* = 272	84/272	55/84 (65.47%)	12/84 (14.28%)	5/84 (5.95%)	50/84 (59.52%)	7/84 (8.33%)	47/84 (55,95%)	2/84 (2,38%)	10/84 (11,9%)		2/84 (2,38%)	6/84 (7,14%)

CMCA: Cefotaxime-supplemented MacConkey Agar

*number and percentage of isolates

In Groups 1, 2, 3, and 4, the distribution of phenotypically ESBL-positive isolates was 32 (66.6%), 13 (65%), 8 (80%), and 2 (33.3%), in that order. Conversely, in Groups 1, 2, and 4, the isolates exhibiting phenotypic AmpC positive were distributed as follows: 9 (18.8%), 1 (5%), and 2 (33.33%). Five *E. coli* isolates exhibited coexistence phenotype of both ESBL and AmpC, with four from Group 1 and one from Group 4.

No carbapenem resistance was detected in any of the *E. coli* isolates (*n* = 212) as a result of the Modified Hodge Test.

### Data regarding the species-wise phenotypic characterization of the beta lactam resistance of *E. coli* isolates

In terms of animal-based evaluation, phenotypic antimicrobial resistance was detected in 59 out of 272 birds (21.7%). Among these, 47 birds exhibited only the ESBL phenotype, 6 displayed only the AmpC phenotype, and 6 showed co-resistance for both types. The majority of resistant birds were categorized within Group 1, with gulls (29 *Larus argentatus*, 5 *Hydrocoloeus minutus*) and one *Ardea cinerea* being particularly prominent. Within this group, beta-lactam resistance was identified in 34 out of 59 gulls (57.6%). In Group 2, resistance was determined in 14 birds, with a predominance of the ESBL phenotype. Group 3 showed ESBL positivity in 7 birds, while resistance was detected in only 3 birds in Group 4.

### Characterisation of beta lactam resistance genes

Genotypic analysis revealed the presence of β-lactamase-encoding genes in 61 out of 84 *E. coli* isolates cultured on cefotaxime-supplemented MacConkey agar. Among these, 51 isolates carried ESBL genes, including *bla*_CTX−M_ (*n* = 50) and *bla*_SHV_ (*n* = 2), while 8 harbored AmpC-associated genes such as *bla*_MOX_ and *bla*_CIT_. Notably, 12 phenotypically ESBL-negative isolates were genotypically positive, and 8 of 12 AmpC-positive isolates were confirmed at the genetic level. No genotypic evidence of AmpC was found among phenotypically negative isolates. Additionally, none of the 212 isolates grown on non-supplemented MacConkey agar carried carbapenemase genes. Distribution by group revealed the highest frequency in Group 1, where 28 birds -including Herring gulls, Little gulls, and a grey heron- harbored resistance genes. Detected genes included *bla*_CTX−M_ (*n* = 25), *bla*_SHV_ (*n* = 1), *bla*_OXA−10_ (*n* = 6), *bla*_MOX_ (*n* = 5), and *bla*_CIT_ (*n* = 1). Seven isolates carried both ESBL and AmpC genes. In Group 2, resistance genes were found in 15 birds, mostly pigeons. Detected genes included *bla*_CTX−M_ (*n* = 15), *bla*_SHV_ (*n* = 1), *bla*_OXA−10_ (*n* = 1), and *bla*_MOX_ (*n* = 1). One pigeon and one magpie carried genotypically resistant isolates despite showing negative or partial phenotypic profiles. In Group 3, resistance genes were identified in seven raptors, with *bla*_CTX−M_ (*n* = 5) and *bla*_OXA−10_ (*n* = 2) detected. No AmpC genes or co-occurrence of resistance determinants were observed. In Group 4, resistance was identified in five birds, including starlings, squacco herons, and a pygmy cormorant. Genes detected were *bla*_CTX−M_ (*n* = 5), *bla*_OXA−10_ (*n* = 1), and *bla*_CIT_ (*n* = 1). Co-occurrence of ESBL and AmpC genes was found in isolates from the pygmy cormorant and one squacco heron. A summary of gene distribution across groups and bird species is presented in Table 3.

**Table 3:** Species-wise Beta Lactam Resistance Profiles of the Study’s Bird Population

Groups	Bird Species with BLR*	n1	Total no & %	Phenotypic			n2	Total no & %	Genotypic ESBL			Genotypic AmpC		AB	
				ESBL	AMPC	ESBL &AMPC			CTX-M	SHV	OXA-10	CIT	MOX	A	B
1	Herring Gull	29	35 (51.5%)	22	3	3	23	28 (41.7%)	21	1	6	1	3	3	5
	Little Gull	5		3	1	1	4		3	-	-	-	1	-	-
	Grey Heron	1		1	-	-	1		1	-	-	-	-	-	-
2	Pigeon	9	14 (20.6%)	9	-	-	12	15 (22%)	12	1	1	-	-	-	1
	Dove	2		2	-	-	1		1	-	-	-	-	-	-
	Crow	2		2	-	-	1		1	-	-	-	-	-	-
	Magpie	1		-	1	-	1		1	-	-	-	1	1	-
3	Owling	2	7 (10.3%)	2	-	-	1	7 (10.3%)	1	-	-	-	-	-	-
	Hawk	3		3	-	-	4		2	-	2	-	-	-	-
	Falcon	2		2	-	-	2		2	-	-	-	-	-	-
4	Starling	1	3 (4.4%)	1	-	-	2	5 (7.3%)	2	-	-	-	-	-	-
	Pygmy cormorant	1		-	1	-	1		1	-	-	1	-	1	-
	Squacco Heron	1		-	-	1	2		2	-	1	-	-	-	1

*: Beta Lactam Resistance; n 1: the number of birds with phenotypic BLR; n2: the number of birds with genotypic BLR

AB: coexistence of ESBL and/or AmpC; A: coexistence of ESBL and AmpC; B: coexistence of ESBL and SHV

### Statistical Data

Among the *E. coli* isolates evaluated, the overall prevalence of phenotypic and genotypic resistance was determined to be 23.5% and 21%, respectively. When analyzed by study groups, the phenotypic and genotypic resistance rates were as follows:

- Group 1: 57.4% (phenotypic), 41.2% (genotypic)
- Group 2: 20.6% (phenotypic), 23.5% (genotypic)
- Group 3: 11.8% (phenotypic), 10.3% (genotypic)
- Group 4: 4.4% (phenotypic), 8.8% (genotypic)

Across all isolates, the antibiotic resistance positivity rate was 23.5%, compared to a negativity rate of 76.5%, a difference that was found to be statistically significant (*p* < 0.001). To compare the presence of phenotypic and genotypic resistance between groups, Chi-square tests were employed, with Fisher’s exact test utilized where expected cell counts were below 5. Group 1 vs. Group 2: Phenotypic positivity rates were 57.4% and 20.6% (*p* < 0.001), while genotypic rates were 41.2% and 23.5% (*p* = 0.028), both indicating significant differences. Group 1 vs. Group 3: Significant differences were observed for both phenotypic (57.4% vs. 11.8%, *p* < 0.001) and genotypic (41.2% vs. 10.3%, p *< 0.001*) resistance. Group 1 vs. Group 4: Phenotypic (57.4% vs. 4.4%, *p* < 0.001) and genotypic (41.2% vs. 8.8%, *p* < 0.001) resistance rates differed significantly. Group 2 vs. Group 3: No significant difference was found in phenotypic resistance (20.6% vs. 11.8%, *p* = 0.162), whereas the difference in genotypic resistance was significant (23.5% vs. 10.3%, p *= 0.040*). Group 2 vs. Group 4: Significant differences were identified for both phenotypic (20.6% vs. 4.4%, *p* = 0.004) and genotypic (23.5% vs. 8.8%, *p* = 0.020) resistance. Group 3 vs. Group 4: No significant differences were observed for either phenotypic (11.8% vs. 4.4%, *p* = 0.104) or genotypic (10.3% vs. 8.8%, *p* = 0.771) resistance.

After applying the Bonferroni correction, significant differences in phenotypic antibiotic resistance were maintained for the comparisons between Group 1 vs. Group 2, Group 1 vs. Group 3, Group 1 vs. Group 4, and Group 2 vs. Group 4. Regarding genotypic resistance, the differences between Group 1 vs. Group 3 and Group 1 vs. Group 4 remained highly significant.

## Discussion

Antimicrobial resistance is a complex and growing global problem affecting humans, animals, and the environment, and its emergence cannot be explained solely by antimicrobial use in clinical or production settings. Increasing evidence indicates that wildlife, including bird populations not directly exposed to antibiotics, may still participate in the environmental circulation and dispersal of resistant bacteria and resistance determinants [7]. In this context, wild birds have attracted particular attention because of their mobility, ecological diversity, and frequent contact with anthropogenically contaminated habitats. Their potential to disseminate resistant microorganisms across large geographic areas during migration and daily foraging has been emphasized in several studies [5, 6].

In the present study, birds living in closer association with human settlements and waste-contaminated environments appeared to play a more prominent role in the carriage of ESBL and/or AmpC producing *E. coli*. This observation is in agreement with previous reports showing that aquatic and urban-associated wild birds, particularly gulls, may act as important carriers of resistant Enterobacterales. Studies conducted in Portugal, France, Florida, Australia, and along the European coastline have repeatedly demonstrated that gulls feeding at landfills, sewage-impacted coastal areas, beaches, and other human-affected habitats frequently harbor resistant *E. coli*, including ESBL producers [8, 10, 11, 16, 20, 21]. The predominance of gulls among positive birds in our study supports this ecological pattern and suggests that the resistant strains or resistance genes detected in these birds are likely linked to environmental exposure originating from human and/or animal sources.

The findings obtained from terrestrial urban birds also support the growing body of evidence indicating that city-dwelling wild birds may contribute to the maintenance and spread of antimicrobial resistance. Pigeons, doves, crows, and similar synanthropic birds are not generally considered direct targets of antimicrobial treatment, yet they often live in close contact with human refuse, animal waste, contaminated water, and heavily populated public spaces. Previous studies have suggested that such birds may serve as reservoirs, sentinels, or mechanical disseminators of resistant bacteria in urban ecosystems [22, 23]. Our results are consistent with these observations and further indicate that urban wild birds should not be viewed merely as passive environmental indicators, but also as biologically relevant components of the resistance cycle.

Compared with resident waterbirds and terrestrial urban birds, migratory bird groups in this study showed a more limited resistance burden. This does not suggest that migratory birds are epidemiologically unimportant; rather, it highlights that resistance carriage may vary according to ecological behavior, feeding strategy, stopover habitats, and degree of contact with anthropogenically contaminated environments. Previous studies from Canada, Pakistan, Bangladesh, Spain, the Netherlands, Alaska, and Tunisia have shown that ESBL and AmpC producing E. coli may also be detected in migratory birds, although the reported prevalence differs considerably depending on the flyway, region, and environmental context [9, 10, 24–26] The lower detection in our migratory groups may therefore reflect reduced or less sustained exposure to contaminated urban niches when compared with resident birds more consistently associated with human-derived waste.

From a genotypic perspective, the predominance of *bla*_CTX−M_ among ESBL associated genes is consistent with the global epidemiology of resistance in *E. coli*. CTX-M-type β-lactamases have become the dominant ESBLs in both human and veterinary medicine, and they are widely reported in environmental and wildlife-associated isolates as well. The detection of *bla*_CTX−M_, particularly *bla*_CTX−M−1_ group genes, in the present study is also in line with reports from Turkey and other regions showing that these genes are widespread in isolates originating from humans, livestock, poultry, companion animals, and wildlife [12, 13, 27]. The additional detection of *bla*_SHV_, *bla*_OXA10_, *bla*_MOX_, and *bla*_CIT_ further indicates that the resistance profiles identified in these birds are genetically diverse and cannot be attributed to a single resistance mechanism.

An interesting finding of the present study was the absence of *bla*_CTX−M−15_, which has been reported frequently in wild birds and environmental isolates from Europe and Africa [19]. This difference may reflect regional variation in circulating resistance gene pools, local ecological conditions, host species composition, or differences in environmental exposure patterns. Rather than representing a contradiction, this finding may indicate that the resistance ecology of wild birds in the Marmara Region has its own local characteristics. It also underlines the importance of region-specific surveillance data, as the dissemination dynamics of antimicrobial resistance genes may differ considerably across ecosystems and geographic settings.

Beyond microbiological findings, the present study has implications for public health and environmental surveillance. Wild birds living in urban coastal areas, parks, landfill-associated habitats, and sewage-influenced environments may facilitate the movement of resistant bacteria between environmental compartments. Through fecal shedding, these birds may contaminate water, soil, recreational areas, animal habitats, and possibly food-related environments [11, 22]. For this reason, the detection of ESBL and/or AmpC producing *E. coli* in wild birds should be considered not only a veterinary or ecological observation, but also a potential indicator of broader environmental exposure and zoonotic risk. At the same time, caution is needed in interpretation. The presence of resistant *E. coli* in wild birds does not by itself demonstrate direct transmission to humans. More detailed epidemiological and genomic studies are needed to clarify the direction, frequency, and significance of such transmission pathways.

Another important contribution of this study is that it provides regional data from Turkey on a relatively underexplored component of antimicrobial resistance ecology. While numerous studies in the country have documented β-lactamase genes in human, livestock, poultry, and companion animal isolates, data from wild birds remain limited [12, 13, 18, 27]. In this respect, the present findings support the inclusion of wild bird populations in integrated surveillance strategies. Species such as gulls and pigeons, particularly those inhabiting densely populated urban environments, may be useful as sentinel hosts for monitoring environmental antimicrobial resistance.

Taken together, the present findings support the One Health view that antimicrobial resistance is maintained through interconnected human, animal, and environmental pathways. Wild birds appear to be part of this interface, especially in ecosystems shaped by human activity. Future studies integrating wild bird sampling with wastewater, landfill, coastal water, and livestock-associated environmental samples, together with whole-genome sequencing and plasmid analysis, would help clarify the circulation routes and epidemiological relevance of resistance determinants in the region.

## Conclusion

This study demonstrated the presence of ESBL and/or AmpC producing *E. coli* in wild birds residing in or seasonally passing through the Marmara Region. The findings indicate that birds more frequently exposed to human activity, animal-associated environments, and waste-contaminated habitats may carry resistant *E. coli* more often than birds with more limited contact with such settings.

The predominance of *bla*_CTX−M_-associated resistance supports the view that wild birds are involved in the broader environmental circulation of clinically relevant resistance genes. In particular, gulls and other urban-associated bird species appear to deserve attention as potential sentinels and carriers within the antimicrobial resistance cycle.

Overall, these results highlight the importance of including wild bird populations in One Health-based antimicrobial resistance surveillance. Monitoring wildlife alongside human, animal, and environmental compartments may provide a more realistic understanding of resistance dissemination and help strengthen public health-oriented control strategies.

## Data Availability

Datasets generated during the current study are available from the corresponding author on request.
